# Estimate of the Costs Caused by Adverse Effects in Hospitalised Patients Due to Hip Fracture: Design of the Study and Preliminary Results

**DOI:** 10.3390/geriatrics3010007

**Published:** 2018-02-15

**Authors:** David Cuesta-Peredo, Francisco Jose Tarazona-Santabalbina, Carlos Borras-Mañez, Angel Belenguer-Varea, Juan Antonio Avellana-Zaragoza, Francisco Arteaga-Moreno

**Affiliations:** 1 Department of Quality Management, IV Hospital Universitario de la Ribera, 46600 Alzira, Valencia, Spain; 2 Department of Geriatrics, Hospital Universitario de la Ribera, 46600 Alzira, Valencia, Spain; fjtarazonas@gmail.com (F.J.T.-S.); ABelenguer@Hospital-Ribera.com (A.B.-V.); javellana@me.com (J.A.A.-Z.); 3 Departamento de Salud de Denia, Atencion Primaria, 03700 Denia, Spain; carlosborras89@gmail.com; 4 Facultad de Medicina, Universidad Catolica de Valencia San Vicente Martir, 46001 Valencia, Spain; francisco.arteaga@ucv.es; 5 C/Baixada Magraners 33, Carcaixent, 46740 Valencia, Spain

**Keywords:** hip fracture, CMBD, DRG, health costs, adverse effects, risk factors, personal history, health quality, patient safety

## Abstract

Introduction: Hip fracture is a health problem that presents high morbidity and mortality, negatively influencing the patient’s quality of life and generating high costs. Structured analysis of quality indicators can facilitate decision-making, cost minimization, and improvement of the quality of care. Methods: We studied 1571 patients aged 70 years and over with the diagnosis of hip fracture at Hospital Universitario de la Ribera in the period between 1 January 2012 and 31 December 2016. Demographic, clinical, functional, and quality indicator variables were studied. An indirect analysis of the costs associated with adverse events arising during hospital admission was made. A tool based on the “Minimum Basic Data Set (CMBD)” was designed to monitor the influence of patient risk factors on the incidence of adverse effects (AE) and their associated costs. Results: The average age of the patients analysed was 84.15 years (SD 6.28), with a length of stay of 8.01 days (SD 3.32), a mean preoperative stay of 43.04 h (SD 30.81), and a mortality rate of 4.2%. Likewise, the percentage of patients with AE was 41.44%, and 11.01% of patients changed their cost as a consequence of these AEs suffered during hospital admission. The average cost of patients was €8752 (SD: 1,864) and the average cost increase in patients with adverse events was €2321 (SD: 3,164). Conclusions: Through the analysis of the main clinical characteristics and the indirect estimation of the complexity of the patients, a simple calculation of the average cost of the attention and its adverse events can be designed in patients who are admitted due to hip fracture. Additionally, this tool can fit the welfare quality indicators by severity and cost.

## 1. Introduction

The factors that affect care quality (CQ) of a healthcare process are numerous (e.g., accessibility, fairness, effectiveness, efficiency, and satisfaction). For this reason, the objective of CQ is a result of positive care for the patient with a maximum level of recovery and in surroundings devoid of adverse events related to medical care.

In this context, in 1999 the US Institute of Medicine (IOM) published the study “To err is human: Building a safer health system” [1] providing relevant data on the magnitude and consequences of adverse events to health care. From this initial study, research expanded to clinical safety. Thus, international organisations (Agency for Healthcare Research and Quality (AHRQ) [2], Organisation for Economic Cooperation and Development (OECD) [3], and the European Economic Community (EEC) [4]) headed by the WHO [5] promoted policies on patient safety, generating studies on the incidence of adverse events, real costs caused by them, and measures to be implemented in order to reduce their number. In addition, indicators were defined that allowed one to compare the results of medical care. In Spain, both the Ministry of Health [6,7] and many of the regions (Observatory of the Health System of Catalonia [8], Observatory of the Results of the Madrilenian Service of Health [9]) headed the implementation of these policies.

Obtaining these measures is difficult due to the characteristics that are intrinsic to the care act, to their complex interpretation, and the presence of confounding factors that lead to adjustments of the measure [7]. 

In the same way, care quality is also dedicated to estimate the economic cost related to the clinical processes. Poor care quality increases costs. Therefore, its quantification facilitates the determination of the amount to invest in order to implement the necessary procedural changes that allow the improvement of clinical practice [10,11,12,13].

One of the clinical processes where CQ has influenced positively is hip fracture. Between 1980 and 2025, the incidence of hip fracture will multiply by 1.84. It is, thus, a health problem of first magnitude that associates high morbidity and mortality, high risk of functional loss, and a considerable increase in healthcare and social costs [14,15,16,17,18,19,20].

The Minimum Data Basic Set (CMBD per Spanish initials) of hospitalised patients is a standardised database with predefined variables promoted by WHO which most Western countries are joining [21,22,23]. The data come from a clinical record and are obtained upon patient discharge. The importance of the CMBD is determined by the need to have homogeneous, uniform, and sufficient sources of data that make the hospital management processes possible, the implementation of new financing systems, the elaboration of performance and use indicators, the control of quality care and patient safety [24], and the availability of information for clinical and epidemiological research [12,13,25,26,27,28,29,30,31,32]. The CMBD gathers demographic data, main diagnosis, risk factors, comorbidity, and adverse events that the patient shows during admission (secondary diagnoses), relevant diagnostic techniques, and therapeutic interventions, above all surgical ones, that have been used to treat the patient. 

The diagnoses and procedures selected in the CMBD are coded following the International Classification of Illnesses (CIE9MC), in its ninth clinical modification (since January 2016 the CIE10MC is used) [33].

With the data of the CMBD, each episode of hospitalization is classified in a diagnostic related group (DRG). Each patient is assigned to a single DRG by specific software, called Grouper, based on a series of well-known and published rules. Each DRG is assigned a relative weight [25,34,35] in terms of the “anticipated cost” for that patient. 

There is no single system of DRGs, and the grouping rules vary throughout time in different versions that are increasingly more adapted to their purpose. From 2012, the version APrDRG (All Patients Refined) [21,35,36,37] of DRGs is available. In Spain, the current official version from January of 2016 is APr32.

In Spain, all national hospitals are forced to register for the CMBD since the early 1990s by a ministerial norm. The CMBD of all patients taken care of in each hospital of the country is recorded [22]. Furthermore, internationally [38], all countries in our socioeconomic surroundings have a CMBD similar to the Spanish one, which allows their use in the policies and strategies of comparative measurement based on them.

The objective of this study was to investigate the prevalence of diseases present at admission and the incidence of adverse events during hospitalization in elderly patients hospitalized for hip fracture. Likewise, the cost associated with these diseases and adverse events was estimated using the rates published by the Spanish Ministry of Health (SMH) for inter-centre billing as a reference. This increase in costs associated with the diseases present at admission, and adverse events that occurred during admission, justifies economic investments to reduce adverse events.

## 2. Methodology

The Hospital Universitario de la Ribera is a third-level hospital which provides care to the population in the area of La Ribera (Valencia) and consists of 256,090 inhabitants, 13.5% of whom are older than 69. The hospital has a Geriatrics Service which consists of five doctors that assist hospitalised elderly patients in the areas of medicine and surgery. In surgery, hospitalised patients older than 69 years are treated due to hip fracture.

During initial evaluation after hospital admission, the traumatologist evaluated the patient and decided the suitability of the surgical treatment and the technique used and the geriatrician carried out a comprehensive geriatric assessment (CGA), including the evaluation of the functional, mental, and social sphere. Additionally, a valuation of the comorbidity and the clinical situation upon admission was conducted, establishing a therapeutic plan during the preoperative period. In the cases when the geriatrician deemed it necessary, the social worker examined the social network of the patient and advised the measures to strengthen it after discharge. The traumatologist and the geriatrician supervised the evolution of the patient daily. 

The model designed aimed to contribute comprehensive and early care, emphasising urgency in geriatric valuation, surgery, and the early beginning of the rehabilitation process in order to recover mobility in the shortest time possible after surgery [20,37,38,39,40,41,42].

An observational, analytical, retrospective study was designed with patients 70 years or older who were receiving care due to code CIE 820** in any of the diagnostic positions of the CMBD at Hospital Universitario de La Ribera between 1 January 2012 and 31 December 2016. The number of patients included in this period was 1,571. 

A total of 175 variables were obtained from each one of the patients studied, of which 106 variables garnered knowledge of their demographic characteristics (age, gender, residence, etc.), their administrative characteristics (date and type of admission date and type of discharge, stays, death, etc.), and their casuistry (diagnoses and procedures related to their care). Sixty-nine variables were obtained from each one of the diagnoses associated with each patient, which allowed us to classify them according to whether it is a diagnostic-therapeutic procedure or a secondary diagnosis corresponding to something in the personal background or an adverse event. 

For the classification of comorbidities, the classification of the groups of diagnoses was used to calculate the Charlson Comorbidity Index [43]. For the classification of the adverse events and/or complications we used CIE9. 

During the coding process, a team of technicians in health documentation reviewed the medical history of the patient and generated the corresponding CMBD. 

In this work, we used the APr32 version of the DRGs currently in effect in Spain. Its main characteristics are:

- Each episode is assigned an SOI (severity of illness) from 1 (not severe) to 4 (very severe).
- Each episode is assigned an ROM (risk of mortality) from 1 (low risk of mortality) to 4 (high risk of mortality).
- The relative weight of each DRG-APr is calculated for each combination of DRG/SOI which allows weights and costs much more adjusted not only to the pathology of the patient, but to his/her previous level of disease (comorbidity) and to its severity.
- At the end of 2015, the Ministry of Health and Consumption published a list of estimated relative weights and costs for each of the pairs of DRG/SOI [44].

The latest versions of this DRGs version incorporate, in addition, the concept present on admission (POA) [45], which tells us whether the diagnosis was present at the time of admission (comorbidity) or was acquired during the hospital stay (adverse events). 

The system APr32-DRG calculates two DRGs for each patient:

- The “GRD on discharge”, which is calculated at the time of hospital discharge for the patient with all diagnoses and procedures coded in the CMBD.
- The “GRD on admission”, which is calculated with the information available at the time of admission, not using the diagnoses POA = NO that reflect the adverse effects during hospitalization.

This work methodology provides the possibility of considering the costs of the adverse events in the patients hospitalized through the differences of weight and severity of both DRGs and based on the standard weights-costs defined by the Ministry of Health.

Not all POA = NO diagnoses cause a change in the DRG and/or severity of the patient (weight/cost); the diagnoses that do are marked with a flag (Affect_SOIFlag) that will allow us to identify them in the phase of analysis.

For each patient, the following variables are calculated:

- On the one hand, all secondary diagnoses whose variable POA is equal to “No” are added up and a discrete quantitative variable (NumPOAs) is obtained with the number of adverse effects that the patient has had during their hospitalization.
- On the other, the previous variable is simplified to know if the patient has or has not had any adverse effect, thus creating the dichotomous qualitative variable nPOA.
- In the same way, we add all secondary diagnoses with variable POA equal to “N” and with variable AffectSOIFlag = “1”. The result is a discrete quantitative variable (NumPOAs_AffectSOIFlag) with the number of adverse effects that affect the change in severity of the patient’s DRG.
- The previous variable is simplified into a qualitative dichotomous one nPOA_AffectSOIFlag as a function of the presence of new diagnoses that produce changes in severity and costs.
- The variable nPOA_Cost (dichotomous/binary) that will have a value = 1 if these adverse effects have resulted in a change in patient DRG and, therefore, in their relative weight and in their cost. The value will be 0 if, in spite of having suffered some adverse effect, it has not had consequences in the assignation of DRG and relative weight.
- The variable CostAPr (continuous quantitative) with the cost/tariff was calculated for the DRG-APr on patient discharge.
- The variable CostAPr_Adm (continuous quantitative) with the cost/tariff was calculated for the DRG-APr at the time of patient admission, not considering adverse effects (POA = NO).
- The variable Diff_Cost_APr (continuous quantitative) with the cost/tariff was calculated for the difference between the DRG-APr at discharge and the DRG-APr at the time of patient admission.

The CMBD-GRDs system incorporates optional added software, potentially preventable complications (PPCs). The PPC system identifies hospital adverse events among the POA = NO, and from them, using an algorithm, classifies them as preventable/non-preventable and assigns them to a determined category.

### 2.1. Statistical Analysis

A description of the qualitative variables (including dichotomous ones) was conducted by means of the use of absolute and relative frequencies. For the quantitative variables, we used measures of central tendency (mean and median), and measures of position (median and quartiles), measures of dispersion (interquartile range and standard deviation). Hypothesis contrast tests were carried out for the study of the different variables (Student’s t, χ^2^ and Mann–Whitney), and the contrasts of the hypothesis are all bilateral, with a significance of 5%.

The software used to conduct the different statistical analyses included in this section was fStats 1.0 (Biostatistics and Investigation Department Medicine and Odontology, Faculty Catholic University of Valencia San Vicente Mártir, Valencia, España) and SPSS 23.0 (LIC. Ribera Salud II UTE).

### 2.2. Declaration of Ethical Commitment

The legal requirements and directives of good clinical practice and those of the declaration of Helsinki (the version updated in October 2008 by the World-wide Medical Association on the ethical principles for medical research with human beings) were complied with. The study was authorised by the Committee of Ethics and Research of Hospital Universitario de la Ribera (Ref approval number PI150715).

## 3. Results

The data were analysed from the 1571 patients hospitalised with hip fracture during the period of the study. In patient profiles, we can highlight a mean age of 84 (SD: 6.1), with a prevalence of women (74%), a mean surgical delay of 43 h (SD: 30.8) with a hospital stay of eight days (SD: 3.3). Mortality was 4.2%. The patients presented high complexity, with comorbidity, estimated by means of Charlson Index of 2.4 (SD: 2.3) points. Furthermore, 3.8% of patients had high anaesthetic risk.

The main variables of the patients included in the sample are described in Table 1.

Table 2 shows that 41.4% of the patients presented at least an adverse event, in 25.6% of the same was observed, at least a diagnosis that modified the degree of severity and, in 11%, the adverse events were responsible for a change in the cost of the patient.

The resulting costs of care of the patients of our study, taking into account the tariffs published by the Ministry of Health on its webpage in relation to the DRGs APr32 [44] was €13,749,524.56, of which the excess cost caused by the appearance of adverse effects (POA = NO) was €401,581.11. The average cost of care to these patients was €8,752.08 (SD 1,864.42), whereas the average cost of adverse effects (taking only the patients that changed in cost) was €2,321.28 (SD 3,164.52).

In Table 3 we can observe that 27% of the patients had a background of diabetes mellitus, 16% had dementia, 11.8% were diagnosed with chronic obstructive pulmonary disease, and 11.4% had chronic kidney pathology. Patients with a personal history of heart failure had a significantly higher mortality, mean stay and case-mix index (AverWeigth_APr). Patients with COPD had a higher score in the case-mix index, those with cerebrovascular disease had a longer stay, and patients with chronic kidney disease had a higher score in the case-mix index and higher mortality, while those with hypertension were older and with diabetes are younger. The adverse events of delirium, cardiac and mortality had significantly higher ages. The hospital stay was higher in patients with EA off delirium, cardiac, anaemia, respiratory, surgical infection, and respiratory infection. Surgical delay was greater in those with EA respiratory and in-hospital mortality.

Table 4 shows the costs of patients with different comorbidity and adverse events. The higher cost in the attention of geriatric patients admitted by hip fracture is related to the previous diagnoses of heart failure (€10,313 SD: €3678.6).

During the hospital stay, the adverse events that appeared most frequently were: delirium (15.1%), anaemia (12%), cardiac adverse events (6.4%), and respiratory events (4.3%). The cost is significantly higher in patients with COPD, chronic kidney disease, and hypertension. With regard to EAs, the cost is significantly higher when delirium, anaemia, cardiac, respiratory or digestive AE, urinary infection, or sepsis appear. The difference in cost due to complications is significantly higher in patients with delirium, EA cardiac or respiratory, urinary infection, respiratory infection, pulmonary thromboembolism, or sepsis.

The hospital stay was higher in the patients with personal background of cardiac insufficiency (9.6 days, SD: 5.5) and when adverse events appeared they were tied to surgical infection (25.1 days, SD 7.5) and to respiratory conditions (14.3 days, SD: 7.5). Similarly, mortality was higher before the presence of adverse effects that were digestive (42.9%), respiratory (31.3%), and cardiac events (28.7%).

In the same way, the cost attributable to the adverse effects is higher in the patients who suffer a surgical infection (€3,714.5 SD: 8,227.5) or a cardiac adverse event (€1,653.0 SD: 3,327.6), a respiratory event (€2,794.0 SD: 4,116.8), or a digestive event (€1,572.5 SD: 3,066.3).

One patient presented an infection of the prosthesis and three of them suffered adverse reactions to drugs as adverse events; in no case did these adverse events show statistically significant associations (*p* > 0.05) with the variables of the study (age, stays, cost and cost difference). 

During the 2012–2015 periods, 14 PPCs were detected (0.89% of the patients). Two of these 14 patients died during the hospital admission (14.28%). We do not have this information for the year 2016.

## 4. Discussion

The results obtained after the analysis of the 1,571 patients of our study showed an age, distribution by gender, and estimated average complexity by means of the Charlson index similar to other published studies [43]. It is worth noting that the results show an average stay around eight days, with a pre-surgical delay less than two days, and a hospital mortality around four percent. In this context, the percentage of adverse events detected that generated a change of estimated average cost for this process was low. The data contributed are better than the average for the country as published by the Spanish Ministry of Health (MSE) [6].

On average our patients are older than those published by the MSE [6] and our pre-surgical stay and hospital stay were lower. Similarly, the mortality published by the MSH for the period studied was 4.92%, while that of our sample was 4.20%, which implies a reduction in mortality by 16% compared to the published state average’s [3]. It is possible that part of these results is due to the permanent update of the clinical guide that manages the welfare process in our hospital. This clinical improvement after update of a clinical guide has previously been described. Tak-Win Lau [46] noted a reduction from 6 to 1.5 days of pre-surgical average stay after the implementation of a clinical guide, and a similar study by Gupta described a reduction of 34 to 19.6 days in the hospital stay after the implementation of a multidisciplinary orthogeriatic care unit [47]. In this same line, another study by Suhm analysed the changes undergone in a service after the implementation of a clinical pathway of hip fracture, verifying how the hospital stay and the probability of experiencing adverse events during the hospitalization after it as reduced, without objectifying differences in institutionalisation or in mortality to one year [48]. Similar results were obtained by another study [47] in which the percentage of patients operated was increased in the first 48 h and the average hospital stay was reduced [47] after the introduction of a model of multidisciplinary orthogeriatric management and measures, like the preoperative geriatric assessment; daily geriatric clinical care; and standardised care protocols. 

In the sample studied, personal background factors standout such as diabetes (27.5% of the patients vs. 21.96% in Culler’s [49]), dementia (15.98% vs. 4.62% [49]), COPD (11.84% vs. 0.28% [49]), chronic kidney problems (11.39% vs. 5.64% [49]), ischemic cardiopathy (6.94% vs. 16.01% [49]), congestive cardiac insufficiency (6.56% vs. 5.50% [49]), and CVA (1.85% vs. 5.55% [49]). In fact, the degrees of prevalence are very similar in both studies, except in CI, which is higher in Culler et al. [49] and in the more elevated COPD in our study. 

In other studies, the risk factors detected in the patients are similar, thus Smith et al. [50] describe a Charlson index, ASA 2–3, gender-male, dementia, intra-capsular fracture. Rosso [51] notes dementia, pre-surgical stay, and having two or more comorbidities. Finally, Ireland [52] discusses dementia (22.5%), kidney problems (13.7%), cardiac insufficiency (13.1%), ischemic cardiopathy (10.2%), diabetes (9.7%), respiratory disease (6.3%), and CVA (6.3%). 

In a prospective work [53], Henderson analysed the main present comorbidities in patients who are admitted with a hip fracture and their influence on mortality. Identified as comorbidities that are more frequent were hypertension, diagnosis of dementia, osteoporosis, ischemic cardiopathy, and chronic obstructive pulmonary disease. Two predictive models of mortality were obtained at 12 months of discharge, one based on comorbidities, which included age, CI, and surgical delay, explaining 26% of the variability in mortality. The model of Henderson was based on the adverse events and included age and respiratory adverse events, also explaining 26% of the variability in mortality. The authors described a significant association between the presence of respiratory adverse events and COPD. 

The average cost considered in the care of our patients was €8,752.08, whereas the estimated extra cost in the patients who suffered at least one adverse event that meant a change of cost was €2,321.28. A greater cost is observed in the patients with personal background of cardiac insufficiency, cerebrovascular diseases, and chronic kidney pathology. Similarly, the cost is greater in those that show adverse effects of surgical, cardiac, or respiratory infection, or digestive or respiratory adverse events.

In this respect, Aigner [54] published a study, with care results very similar to ours, of a prospective cohort of 402 patients with hip fracture. In this study, an analysis of the factors associated with the cost increase of hospital care was carried out. In the estimated calculations, the average cost by patient was €8,853 (SD 5,676) of which €5,288 (SD 4,294) were in hospitalization room costs and €1,972 (SD 956) in operating room costs. The authors concluded the article indicating the need to establish payment systems adjusted to the specific risks of these patients. In the same way, Culler et al. [49] published a study in 2017 on the increase of the hospital cost involved in the adverse events between the beneficiaries of the programme available during tax year 2014. Its cost varied widely from $6,308 to $29,061 based on the number and type of detected adverse effects. Adverse effects studied were: death, acute infarct of myocardium, pneumonia, sepsis, shock, surgical haemorrhage, pulmonary embolism, and prosthetic joint infection. 

Other studies have approached the analysis of costs from other aspects. Thus, Nichols [55] analysed the costs in the process of arthroplasty (stratified in four different DRGs) in the 90 days after surgery with an average of $28,952, $19,243, $29,763, and $18,561 in each of the groups.

In another study [56], Ginsberg conducted a study of cost/usefulness that compared a model of orthogeriatric care with respect to a reactive orthogeriatric service, establishing that orthogeriatric care presented cost/effectiveness. The orthogeriatric model of care used 23% fewer resources by patient ($14,919 vs. $19,363) and avoided 0–226 disability-adjusted life years (DALYs) by patient, adding years of quality of life adjusted when reducing the cost of institutionalization by patient, reducing mortality to one year [56]. A retrospective study of cohorts compared the orthogeriatric care with respect to habitual traumatological care, finding an average of $13,737 by patient and a reduction in mortality at 12 months (Della Rock in 2013) [57]. 

Finally, a prospective study of randomized intervention compared the attention in an orthogeriatric unit based on the care in a room of orthopedic surgery with respect to the geriatric care by interconsultation and found that the patients taken care of in the orthogeriatric unit had a greater probability of initiating rehabilitation in the acute room, a greater recovery of the capacity to ramble, earlier surgery and a shorter hospital stay. This meant a savings of €1,207–€1,633 in cost by patient considered of the process and €3,741 when the costs by avoided stays were considered (Gonzalez Montalvo in 2011) [58].

The main limitations of this study were their retrospective character and the low sensitivity of the diagnoses. One of the problems of the retrospective analyses is the heterogeneity of the quality of the data in the medical histories (Barba et al. [29]). One of the obtained conclusions of this limitation is the possibility of qualifying specific electronic items that allow improving the analysis of the quality indicators; thus, transfusional levels have already been added to the data on pre- and post-haemoglobin analysis and data of execution of functional scales (e.g., the Barthel Index or the Lawton Instrumental Activities of Daily Living Scale) that will allow these data to have an automated form. Through the analysis of the main clinical characteristics and the indirect estimation of patient complexity, a simple calculation of the average cost of care and its adverse events can be designed in patients who are admitted due to hip fracture. Furthermore, this tool can adjust the quality care indicators by severity and cost. In this manner, we can obtain the average cost of patients classified by different measurements of severity/complexity and according to surgical delay. This tool facilitates the monitoring of the quality of any care process.

## Figures and Tables

**Table 1:** Sample characteristics.

Variables		*n* = 1571
Age (years)		84.0 (SD: 6.1)
Sex	Male	408 (25.97%)
	Female	1163 (74.03%)
Surgical delay (hours)		43.0 (SD: 30.8)
	Delay < 48 h	1038 (66.07%)
Hospital stay (Days)		8.0 (SD: 3.3)
	Stays < 10 Days	1376 (87.59%)
Mortality		66 (4.2%)
Fracture Type	Intracapsular	907 (57.73%)
	Extracapsular	660 (42.02%)
	Other	4 (0.25%)
Surgery Type	Intracapsular	1014 (64.55%)
	Extracapsular	527 (33.55%)
	Other	30 (1.90%)
Anaesthesia Type	Rachidian	1302 (82.88%)
	General	222 (14.13%)
	Other	47 (2.99%)
ER admission		16 (1.02%)
Charlson Index		2.4 (SD: 2.3)
APrSev (SOI)	1	652 (41.50%)
	2	746 (47.49%)
	3	150 (9.55%)
	4	23 (1.46%)
APrMort (ROM)	1	702 (44.68%)
	2	711 (45.26%)
	3	133 (8.47%)
	4	25 (1.59%)
ASA	0	5 (0.32%)
	1	416 (26.48%)
	2	754 (47.99%)
	3	60 (3.82%)
AverWeight_Apr		1.8284 (SD: 0.3895)

Legend: Age = age of the patient at the time of hospital admission. Surgical delay = hours of delay from admission to surgery. Hospital stay = days of patient stay in the hospital. Mortality = % of patients who pass away during hospitalisation. Fracture type = anatomical location of the fracture. Surgery type = type of surgical technique used. Anaesthesia type = type of anaesthetic technique used. ER admission = number of patients who needed admission into the emergency room. Charlson Index = measurement of patient comorbidity. APrSEV = severity of illness (level). APrMort = risk of mortality (level). ASA = measurement of anaesthetic risk (level). AverWeight_APr = relative weight or case mix of the patient (grouping APr32-DRG).

**Table 2:** Adverse events and associated costs.

Variables	*n* = 1,571
nPOA	651 (41.4%)
nPOA_AffectSOIFlag	402 (25.6%)
nPOA_Cost	173 (11.0%)
Cost_Apr (Total in Euros) annual	€13,749,524.6
Cost_Apr (Mean and SD in Euros)	€8,752.1 (SD:1,864.4)
Diff_Cost_Apr (Total in Euros) annual	€401,581.1
Diff_Cost_Apr (Mean and SD in Euros)	€2,321.3 (SD: 3,164.5)

Legend: nPOA = number of patients with adverse events. POA_AffectSOIFlag = number of patients with change in severity. nPOA_Cost = number of patients with change in cost. Cost_APr = cost of patient in Euros according to Ministry of Health tariff. Diff_Cost_Apr = cost attributable to patient adverse events.

**Table 3:** Quality indicators segmented by previous background and adverse effects.

VARIABLES		Discharges	Age (Years)	*p* Value	Hospital Stay (Days)	*p* Value	*n* (%) Mortality	*p* Value	DelayIQ (Hours)	*p* Value	AverWeight_APr	*p* Value
Total Cases		1571	84.1 (SD: 6.3)		8.0 (SD: 3.3)		4.2%		43.0 (SD: 30.8)		1.8284 (SD: 0.3895)	
PREVIOUS DIAGNOSE (PD)												
PD of Ischemic Cardiopathy	Yes	109 (6.9%)	84.0 (SD: 6.1)	0.772	8.4 (SD: 4.1)	0.162	6 (5.5%)	0.455	41.6 (SD: 23.8)	0.602	1.8938 (SD: 0.2837)	0.069
	No	1462 (93.1%)	84.2 (SD: 6.3)		8.0 (SD: 3.3)		60 (4.1%)		43.2 (SD: 31.3)		1.8236 (SD: 0.3959)	
PD of Cardiac Insufficiency	Yes	103 (6.6%)	84.2 (SD: 6.3)	0.941	9.6 (SD: 5.5)	0.003	13 (12.6%)	<0.001	54.9 (SD: 61.4)	0.039	2.1545 (SD: 0.7685)	<0.001
	No	1468 (93.4%)	84.2 (SD: 6.3)		7.9 (SD: 3.1)		53 (3.6%)		42.2 (SD: 27.3)		1.8056 (SD: 0.3366)	
PD of Chronic Obstructive Pulmonary Disease	Yes	186 (11.8%)	83.3 (SD: 6.1)	0.056	8.4 (SD: 3.8)	0.131	12 (6.5%)	0.117	46.9 (SD: 32.7)	0.067	1.9286 (SD: 0.4932)	0.003
	No	1385 (88.2%)	84.3 (SD: 6.3)		8.0 (SD: 3.2)		54 (3.9%)		42.5 (SD: 30.5)		1.8150 (SD: 0.3751)	
PD of Cerebrovascular Disease	Yes	29 (1.8%)	82.7 (SD: 8.1)	0.322	9.4 (SD: 5.3)	0.022	2 (6.9%)	0.346	43.6 (SD: 23.3)	0.919	2.0380 (SD: 0.7277)	0.126
	No	1542 (98.2%)	84.2 (SD: 6.2)		8.0 (SD: 3.3)		64 (4.2%)		43.0 (SD: 30.9)		1.8244 (SD: 0.3796)	
PD of Dementia	Yes	251 (16.0%)	84.7 (SD: 6.0)	0.152	8.0 (SD: 3.0)	0.742	9 (3.6%)	0.732	41.3 (SD: 24.6)	0.339	1.8237 (SD: 0.3093)	0.835
	No	1320 (84.0%)	84.1 (SD: 6.3)		8.1 (SD: 3.4)		57 (4.3%)		43.4 (SD: 31.8)		1.8293 (SD: 0.4030)	
PD of Kidney Disease	Yes	179 (11.4%)	84.5 (SD: 6.5)	0.375	8.7 (SD: 4.4)	0.026	16 (8.9%)	0.002	42.9 (SD: 25.3)	0.957	1.9949 (SD: 0.4679)	<0.001
	No	1392 (88.6%)	84.1 (SD: 6.3)		7.9 (SD: 3.1)		50 (3.6%)		43.1 (SD: 31.5)		1.8070 (SD: 0.3731)	
PD of Diabetes	Yes	432 (27.5%)	83.4 (SD: 5.8)	0.001	8.0 (SD: 3.1)	0.851	16 (3.7%)	0.673	44.7 (SD: 39.8)	0.192	1.8464 (SD: 0.4340)	0.260
	No	1139 (72.5%)	84.5 (SD: 6.4)		8.0 (SD: 3.4)		50 (4.4%)		42.4 (SD: 26.6)		1.8216 (SD: 0.3712)	
PD of Hypertension	Yes	1051 (66.9%)	84.5 (SD: 6.1)	<0.001	7.9 (SD: 3.1)	0.639	45 (4.3%)	0.469	42.4 (SD: 25.8)	0.211	1.8217 (SD: 0.3281)	0.330
	No	520 (33.1%)	83.3 (SD: 6.4)		8.1 (SD: 3.7)		21 (4.0%)		44.4 (SD: 39.0)		1.8420 (SD: 0.4907)	
ADVERSE EFFECTS (AE)												
EA de Delirium	Yes	238 (15.1%)	86.5 (SD: 5.6)	<0.001	8.9 (SD: 4.1)	<0.001	6 (2.5%)	0.218	45.8 (SD: 25.5)	0.139	1.9229 (SD: 0.4153)	<0.001
	No	1333 (84.9%)	83.7 (SD: 6.3)		7.9 (SD: 3.1)		60 (4.5%)		42.6 (SD: 31.6)		1.8116 (SD: 0.3824)	
EA Cardiac disease	Yes	101 (6.4%)	86.1 (SD: 6.4)	0.001	10.8 (SD: 6.0)	<0.001	29 (28.7%)	<0.001	54.3 (SD: 62.1)	0.056	2.1733 (SD: 0.7712)	<0.001
	No	1,470 (93.6%)	84.0 (SD: 6.3)		7.8 (SD: 0.30)		37 (2.5%)		42.3 (SD: 27.4)		1.8047 (SD: 0.3360)	
AE of Anaemia	Yes	188 (12.0%)	84.2 (SD: 6.6)	0.893	9.0 (SD: 4.6)	0.002	11 (5.9%)	0.243	40.0 (SD: 23.7)	0.069	1.8941 (SD: 0.0337)	0.014
	No	1383 (88.0%)	84.1 (SD: 6.1)		7.9 (SD: 3.1)		55 (4.0%)		43.5 (SD: 31.6)		1.8195 (SD: 0.3777)	
AE of Urinary Infection	Yes	50 (3.2%)	83.3 (SD: 6.5)	0.354	10.1 (SD: 4.1)	<0.001	1 (2.0%)	0.721	45.9 (SD: 26.1)	0.502	1.9961 (SD: 0.4166)	0.002
	No	1521 (96.8%)	84.2 (SD: 6.3)		7.9 (SD: 3.3)		65 (4.3%)		43.0 (SD: 31.0)		1.8229 (SD: 0.3875)	
Digestive AE	Yes	7 (0.4%)	87 (IQ: 77–91.5)	0.982	12 (IQ: 9.5–15)	0.099	3 (42.9%)	0.002	25 (IQ: 14.5–38)	0.456	2.1758 (IQ: 1.8588–2.1758)	0.263
	No	1564 (99.6%)	84 (IQ: 80–88)		7 (IQ: 6–9)		63 (4.0%)		34 (IQ: 23–58)		1.7564 (IQ: 1.7564–1.8193)	
Respiratory AE	Yes	67 (4.3%)	85.2 (SD: 6.7)	0.147	14.3 (SD: 7.5)	<0.001	21 (31.3%)	<0.001	64.7 (SD: 85.0)	0.033	2.3831 (SD: 0.9381)	<0.001
	No	1504 (95.7%)	84.1 (SD: 6.3)		7.7 (SD: 2.7)		45 (3.0%)		42.1 (SD: 25.5)		1.8037 (SD: 0.3248)	
AE of Surgical Infection	Yes	10 (0.6%)	86 (IQ: 80–89)	0.728	25 (IQ: 21.5–29.25)	<0.001	1 (10.0%)	0.353	55 (IQ: 18–78.25)	0.593	1.9612 (IQ: 1.9612–3.0556)	<0.001
	No	1561 (99.4%)	84 (IQ: 80–88)		7 (IQ: 6–9)		65 (4.2%)		34 (IQ: 23–57)		1.7564 (IQ: 1.7564–1.8193)	
AE of Respiratory Infection	Yes	23 (1.5%)	87 (IQ: 82–89.5)	0.202	10 (IQ: 9–13.5)	<0.001	5 (21.7%)	0.002	48 (IQ: 23.5–79)	0.065	1.9612 (IQ: 1.8193–2.1758)	<0.001
	No	1548 (98.5%)	84 (IQ: 80–88)		7 (IQ: 6–9)		61 (3.9%)		33 (IQ: 23–56.25)		1.7564 (IQ: 1.7564–1.8193)	
AE of Sepsis	Yes	9 (0.6%)	82 (IQ: 80–84)	0.247	10 (IQ: 8–13)	0.015	3 (33.3%)	0.005	48 (IQ: 25–59)	0.639	1.9612 (IQ: 1.8193–2.1758)	0.404
	No	1562 (99.4%)	84 (IQ: 80–88)		7 (IQ: 6–9)		63 (4.0%)		34 (IQ: 23–57)		1.7564 (IQ: 1.7564–1.8193)	
AE of Shock	Yes	3 (0.2%)	87 (IQ: 80.5–89)	0.842	13 (IQ: 9.5–21.5)	0.179	1 (33.3%)	0.121	59 (IQ: 38–81)	0.505	1.7564 (IQ: 1.7564–2.5526)	0.508
	No	1568 (99.8%)	84 (IQ: 80–88)		7 (IQ: 6–9)		65 (4.1%)		34 (IQ: 23–57)		1.7564 (IQ: 1.7564–1.8193)	
AE of pulmonary embolism	Yes	5 (0.3%)	80 (IQ: 79–86)	0.414	10 (IQ: 7–14)	0.144	2 (40.0%)	0.016	74 (IQ: 52–82)	0.124	1.8193 (IQ: 1.7564–2.1758)	0.406
	No	1566 (99.7%)	84 (IQ: 80–88)		7 (IQ: 6–9)		64 (4.1%)		34 (IQ: 23–57)		1.7564 (IQ: 1.7564–1.8193)	
EA of Surgical haemorrhage	Yes	9 (0.6%)	86 (IQ: 82–86)	0.857	9 (IQ: 8–10)	0.060	2 (22.2%)	0.052	40 (IQ: 26–64)	0.911	1.8193 (IQ: 1.7564–2.1758)	0.194
	No	1562 (99.4%)	84 (IQ: 80–88)		7 (IQ: 6–9)		64 (4.1%)		34 (IQ: 23–57)		1.7564 (IQ: 1.7564–1.8193)	
EA exitus	Yes	66 (4.2%)	87.5 (SD: 7.0)	<0.001	9.1 (SD: 5.7)	0.116			59.0 (SD: 67.4)	0.049	2.2954 (SD: 1.0063)	<0.001
	No	1505 (95.8%)	84.0 (SD: 6.2)		8.0 (SD: 3.2)				42.3 (SD: 28.0)		1.8080 (SD: 0.3234)	

Legend: Age = age of the patient at the time of hospital admission. Hospital stay = days of patient stay in the hospital. Mortality = % of patients who pass away during hospital admission. DelayIQ = hours of delay from Admission to Surgery. AverWeight_APr = relative weight of CaseMix of the patient (buncher APr32-DRG).

**Table 4:** Costs segmented by previous background and adverse effects.

VARIABLES		Cost_APr	*p* Value	nPOA	*p* Value	nPOA_AffectSOIFlag	*p* Value	nPOA_Cost	*p* Value	Diff_Cost_Apr	*p* Value
Total Cases		€8752.1 (SD: 1864.4)		651 (41.4%)		402 (25.6%)		173 (11.0%)		€2321.3 (SD: 3164.5)	
PREVIOUS DIAGNOSE (PD)											
PD of Ischemic Cardiopathy	Yes	€9,065.1 (SD: 1,358.0)	0.069	64 (58.7%)	<0.001	34 (31.2%)	0.173	13 (11.9%)	0.751	€212.1 (SD: 901.2)	0.712
	No	€8,728.7 (SD: 1,895.1)		587 (40.2%)		365 (25.2%)		160 (10.9%)		€258.9 (SD: 1,298.7)	
PD of Cardiac Insufficiency	Yes	€10,313.0 (SD: 3,678.6)	<0.001	58 (56.3%)	0.002	36 (35.0%)	0.027	19 (18.4%)	0.021	€827.9 (SD: 2,979.8)	0.041
	No	€8,642.6 (SD: 1,611.3)		593 (40.4%)		366 (24.9%)		154 (10.5%)		€215.5 (SD: 1,047.7)	
PD of Chronic Obstructive Pulmonary Disease	Yes	€9,231.4 (SD: 2,360.9)	0.003	83 (44.6%)	0.383	53 (28.5%)	0.326	28 (15.1%)	0.079	€447.4 (SD: 1,888.8)	0.127
	No	€8,687.7 (SD: 1,778.5)		568 (41.0%)		349 (25.2%)		145 (10.5%)		€229.9 (SD: 1,166.8)	
PD of Cerebrovascular Disease	Yes	€9,755.3 (SD: 3,483.1)	0.123	16 (55.2%)	0.133	7 (24.1%)	1.000	3 (10.3%)	1.000	€116.1 (SD: 403.6)	0.552
	No	€8,733.2 (SD: 1,817.1)		635 (41.2%)		395 (25.6%)		170 (11.0%)		€258.2 (SD: 1,285.6)	
PD of Dementia	Yes	€8,729.6 (SD: 1,480.7)	0.835	117 (46.6%)	0.081	75 (29.9%)	0.097	29 (11.6%)	0.742	€243.2 (SD: 1,016.2)	0.867
	No	€8,756.4 (SD: 1,929.2)		534 (40.5%)		327 (24.8%)		144 (10.9%)		€258.0 (SD: 1,318.7)	
PD of Kidney Disease	Yes	€9,548.9 (SD: 2,239.8)	0.001	104 (58.1%)	<0.001	70 (39.1%)	<0.001	36 (20.1%)	0.001	€589.5 (SD: 1,904.2)	0.010
	No	€8,649.6 (SD: 1,785.8)		547 (39.3%)		332 (23.9%)		137 (9.8%)		€212.7 (SD: 1,163.8)	
PD of Diabetes	Yes	€8,838.2 (SD: 2,077.2)	0.260	195 (45.1%)	0.075	129 (29.9%)	0.020	55 (12.7%)	0.206	€301.2 (SD: 1,575.4)	0.383
	No	€8,719.4 (SD: 1,776.9)		456 (40.0%)		273 (24.0%)		118 (10.4%)		€238.4 (SD: 1,140.8)	
PD of Hypertension	Yes	€8,719.9 (SD: 1,570.7)	0.003	468 (55.5%)	<0.001	288 (27.4%)	0.011	119 (11.3%)	0.320	€219.1 (SD: 917.3)	0.002
	No	€8,817.2 (SD: 2,348.9)		183 (35.2%)		114 (21.9%)		54 (10.4%)		€329.4 (SD: 1,790.7)	
ADVERSE EFFECTS (AE)											
EA de Delirium	Yes	€9,204.2 (SD: 1,988.4)	<0.001	238 (100.0%)	<0.001	211 (88.7%)	<0.001	67 (28.2%)	<0.001	€602.2 (SD: 1,775.9)	0.001
	No	€8,671.4 (SD: 1,830.5)		413 (31.0%)		191 (14.3%)		106 (8.0%)		€193.7 (SD: 1,153.0)	
EA Cardiac disease	Yes	€10,403.3 (SD: 3,691.5)	<0.001	101 (100.0%)	<0.001	73 (72.3%)	<0.001	55 (54.5%)	<0.001	€1,653.0 (SD: 3,327.6)	<0.001
	No	€8,638.6 (SD: 1,608.4)		550 (37.4%)		329 (22.4%)		118 (8.0%)		€159.6 (SD: 916.48)	
AE of Anaemia	Yes	€9,066.5 (SD: 2,216.0)	0.014	188 (100.0%)	<0.001	116 (61.7%)	<0.001	41 (21.8%)	<0.001	€387.6 (SD: 1,144.8)	0.131
	No	€8,709.3 (SD: 1,808.1)		463 (33.5%)		286 (20.7%)		132 (9.5%)		€237.7 (SD: 1,291.0)	
AE of Urinary Infection	Yes	€9,554.5 (SD: 1,994.2)	0.005	50 (100.0%)	<0.001	44 (88.0%)	<0.001	24 (48.0%)	<0.001	€964.1 (SD: 1,639.3)	0.003
	No	€8,725.7 (SD: 1,854.8)		601 (39.5%)		358 (23.5%)		149 (9.8%)		€232.3 (SD: 1,255.1)	
Digestive AE	Yes	€10,736.6 (SD: 3,203.8)	0.005	6 (85.7%)	0.023	6 (85.7%)	0.001	3 (42.9%)	0.033	€1,572.5 (SD: 3,066.3)	0.297
	No	€8,743.2 (SD: 1,853.2)		645 (41.2%)		396 (25.3%)		170 (10.9%)		€249.7 (SD: 1,260.5)	
Respiratory AE	Yes	€11,407.1 (SD: 4,490.2)	<0.001	67 (100.0%)	<0.001	57 (85.1%)	<0.001	49 (73.1%)	<0.001	€2,794.0 (SD: 4,116.8)	<0.001
	No	€8,633.8 (SD: 1,554.8)		584 (38.8%)		345 (22.9%)		124 (8.2%)		€142.5 (SD: 808.5)	
AE of Surgical Infection	Yes	€13,439.4 (SD: 8,596.1)	0.117	10 (100.0%)	<0.001	6 (60.0%)	0.022	5 (50.0%)	0.002	€3,714.5 (SD: 8,227.5)	0.214
	No	€8,722.1 (SD: 1,711.8)		641 (41.1%)		396 (25.4%)		168 (10.8%)		€233.5 (SD: 1,080.8)	
AE of Respiratory Infection	Yes	€9,433.1 (SD: 1,862.7)	0.078	23 (100.0%)	<0.001	19 (82.6%)	<0.001	15 (65.2%)	<0.001	€1,046.3 (SD: 1,314.9)	0.008
	No	€8,742.0 (SD: 1,863.2)		628 (40.6%)		383 (24.7%)		158 (10.2%)		€243.9 (SD: 1,271.1)	
AE of Sepsis	Yes	€9,388.1 (IQ: 8,708.4–10,414.8)	0.003	9 (100.0%)	<0.001	7 (77.8%)	0.002	6 (66.7%)	<0.001	€679.6 (IQ: 0–2,007.3)	<0.001
	No	€8,407.6 (IQ: 8,407.6–8,708.4)		642 (41.1%)		395 (25.3%)		167 (10.7%)		0 (IQ: 0–0)	
AE of Shock	Yes	€8,407.6 (IQ: 8,407.6–12,218.8)	0.635	3 (100.0%)	0.071	0 (0.0%)	0.412	0 (0.0%)	0.705	0 (IQ: 0–0)	0.543
	No	€8,407.6 (IQ: 8,407.6–8,708.4)		648 (41.3%)		402 (25.6%)		173 (11.0%)		0 (IQ: 0–0)	
AE of pulmonary embolism	Yes	€8,708.4 (IQ: 8,407.6–10,414.8)	0.349	5 (100.0%)	0.012	4 (80.0%)	0.017	3 (60.0%)	0.011	€983.6 (IQ: 0–2,990.9)	<0.001
	No	€8,407.6 (IQ: 8,407.6–8,708.4)		646 (41.3%)		398 (25.4%)		170 (10.9%)		0 (IQ: 0–0)	
EA of Surgical haemorrhage	Yes	€8,708.4 (IQ: 8,407.6–10,414.8)	0.187	9 (100.0%)	<0.001	4 (44.4%)	0.176	2 (22.2%)	0.260	0 (IQ: 0–0)	0.255
	No	€8,407.6 (IQ: 8,407.6–8,708.4)		642 (41.1%)		398 (25.5%)		171 (10.9%)		0 (IQ: 0–0)	
EA exitus	Yes	€10,987.4 (SD: 4,816.9)	<0.001	54 (81.8%)	<0.001	43 (65.2%)	<0.001	34 (51.5%)	<0.001	€2,193.0 (SD: 4,373.2)	<0.001
	No	€8,654.1 (SD: 1,548.2)		597 (39.7%)		359 (23.9%)		139 (9.2%)		€170.7 (SD: 835.6)	

Legend: Cost_APr = cost of the patient in Euros according to the Ministry of Health. nPOA = number of patients with adverse events. POA_AffectSOIFlag = number of patients with change in severity. nPOA_Coste = number of patients with cost change. Diff_Coste_Apr = cost attributable to patient adverse events.
